# Cortical Contributions to Saccadic Suppression

**DOI:** 10.1371/journal.pone.0006900

**Published:** 2009-09-04

**Authors:** George Chahine, Bart Krekelberg

**Affiliations:** Center for Molecular and Behavioral Neuroscience, Rutgers University, Newark, New Jersey, United States of America; Istituto di Neurofisiologia, Italy

## Abstract

The stability of visual perception is partly maintained by saccadic suppression: the selective reduction of visual sensitivity that accompanies rapid eye movements. The neural mechanisms responsible for this reduced perisaccadic visibility remain unknown, but the Lateral Geniculate Nucleus (LGN) has been proposed as a likely site. Our data show, however, that the saccadic suppression of a target flashed in the right visual hemifield increased with an increase in background luminance in the left visual hemifield. Because each LGN only receives retinal input from a single hemifield, this hemifield interaction cannot be explained solely on the basis of neural mechanisms operating in the LGN. Instead, this suggests that saccadic suppression must involve processing in higher level cortical areas that have access to a considerable part of the ipsilateral hemifield.

## Introduction

Humans move their eyes about three times each second. Those rapid eye movements - called saccades – help to increase our perceptual resolution by placing different parts of the world on the high-resolution fovea. As these eye movements are performed, the image is swept across the retina, yet we perceive a stable world with no apparent blurring or motion. One mechanism that has been proposed to account for this stability is saccadic suppression; or the decrease of visual sensitivity during eye movements. The evidence suggests that suppression is mediated by a selective mechanism that dampens motion signals, possibly by targeting the magnocellular pathway [1].

The site of suppression in the brain is still unknown. Some evidence suggests that it is very early in the visual pathway, possibly as early as the lateral geniculate nucleus of the thalamus (LGN). Specifically, Thilo et al showed that phosphenes evoked by transcranial magnetic stimulation (TMS) to the occipital cortex are not suppressed during saccades, while phosphenes evoked by electric stimulation of the retina are suppressed before, during and after saccades[2]. At first sight this evidence seems a rather compelling demonstration that the LGN is the main site of saccadic suppression. Physiological and functional imaging methods, however, indicate that suppression is more complex than a photographic shutter that operates at the level of the thalamus [3]. This discrepancy in the literature led us to our current research.

Anatomically, the optic tracts that innervate the right LGN conduct information coming from the left visual field while those that innervate the left LGN conduct information coming from the right visual field. Physiologically, it has been verified that cells in each LGN respond only to visual stimuli in the respective contralateral visual field [4]. Due to this separation of inputs, perceptual phenomena that rely on the interaction of input from the two hemifields cannot have a purely thalamic or retinal origin and must include at least some processing in cortical areas. We exploited this well-known fact to determine whether mechanisms underlying saccadic suppression rely on cortical mechanisms.

**Figure 1:**
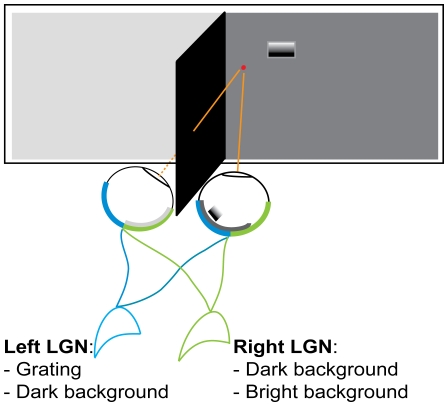
Sketch of the physical setup. Our setup used a physical barrier to ensure that stimuli left of the midline could only reach the right LGN while those to the right of the fixation point could reach only the left LGN. the change of background luminance in the left hemifield could not be detected by the LGN that processed the grating.

Previous studies have shown that saccadic suppression increases with an increase in background luminance [5]. In our experiments we asked whether a background change that is confined to one visual hemifield could influence the saccadic suppression of a target in the contralateral hemifield (Figure 1). By flashing a luminance-modulated grating in the right visual field and changing the background luminance in the left visual field only, we ensured that the left LGN that detects the grating cannot detect the change in background luminance. Consequently, any variation in the saccadic suppression of the grating with the change of background luminance cannot be attributed to the LGN, but must involve cortical cells that receive visual information from both visual fields.

## Results

We found that the background luminance of the left visual hemifield strongly affected the pre-saccadic visibility of a grating presented in the right visual hemifield. We will first present the results of a single observer, followed by an overview of the average effect across all observers.

**Figure 2:**
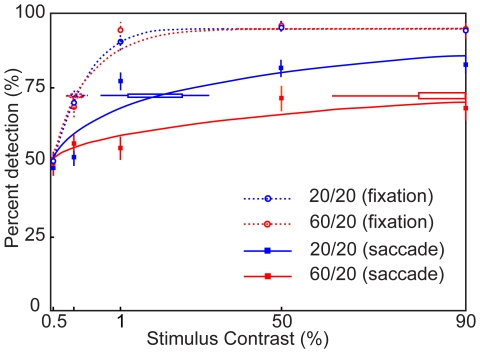
Changes in detection threshold with a contralateral change in background luminance. Performance as a function of the grating contrast, separately for saccade (solid lines) and fixation (dashed lines) conditions, and for the two background luminance conditions. The red curves correspond to the 60/20 condition while the blue curves correspond to the 20/20 condition. Error bars show 95% confidence intervals. An increase in contralateral background luminance did not affect performance during fixation, but it significantly increased the detection threshold for pre-saccadic gratings.

Figure 2 shows the detection performance of one observer for a range of grating contrasts, just before a saccade and during steady fixation, and in the two background luminance conditions. Clearly, performance during fixation (dashed lines) was not affected by the background luminance. Saccadic suppression is evident from the fact that performance in the saccade conditions was reduced at all levels of stimulus contrast. The critical finding for our current study, however, was that the subject's detection threshold (defined as the contrast at which 74% correct performance was attained) increased nearly five-fold when the background luminance of the opposite hemifield was increased from 20 cd/m^2^ to 60 cd/m^2^.

**Figure 3:**
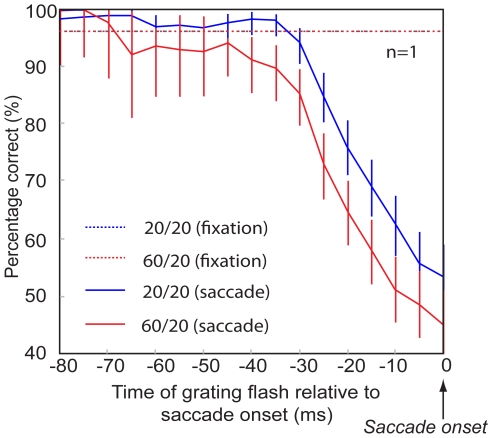
Time course of pre-saccadic detection performance. Each data point represents the percentage correct in a centered 20 ms wide temporal interval. Error bars correspond to 95% confidence intervals calculated from binomial error proportion analysis. Performance was unaffected by the contralateral background luminance during fixation, but a higher contralateral background luminance consistently led to worse performance from approximately 50 ms before saccade onset.

To investigate how this contralateral background influence developed over time, we choose a single grating contrast, and presented that same grating at a wide range of times before saccade onset. Figure 3 shows this time course for a single observer. Performance in the saccade condition for the 20/20 condition was consistently better than performance in the 60/20 condition. No difference in performance between conditions was observed during fixation.

**Figure 4:**
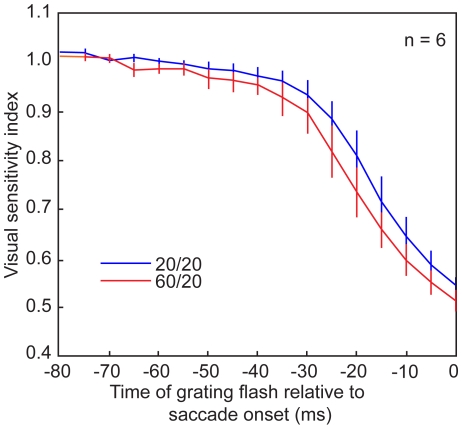
Time course of pre-saccadic visual sensitivity. The visual sensitivity index (see Methods) was averaged across subjects and shown here separately for the two luminance conditions. Error bars correspond to the standard error in the mean. The figure shows that the effect of contralateral luminance changes shown for a single subject in Figure 3 is representative for all our subjects.

To analyze these effects at the group level (N = 6), we computed a visual sensitivity index (see Methods). A two-way RM ANOVA on the visual sensitivity indices for all six subjects revealed main effects of time-to-saccade and background luminance. The main effect of time (p<0.001, F = 163.34) showed the expected decrease in visual sensitivity as the time of the grating flash became closer to saccade onset; i.e. there was significant saccadic suppression. More importantly for our current purpose, the ANOVA also showed a significant main effect of the background luminance (p = 0.0212, F = 10.97). This shows that the change of background luminance in the left hemifield led to an increase in the saccadic suppression of the grating in the right hemifield. Figure 4 shows the time course of the average visual sensitivity index across all subjects in the two luminance conditions.

**Figure 5:**
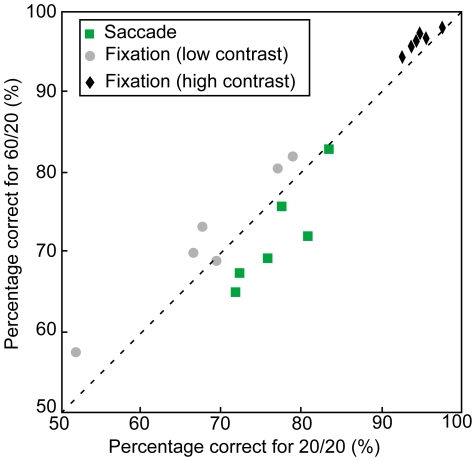
Performance in the two background luminance conditions for all subjects, during fixation and just before an eye movement. Green squares correspond to performance in saccade conditions for a 50% contrast grating flashed just before an eye movement was made. Black diamond shapes correspond to the detection of 50% contrast gratings during fixation. Grey circles correspond to the detection of gratings during fixation with contrast values that varied from subject to subject, such that detection performance was well below ceiling. A sign test revealed an effect of luminance on detection in the saccade condition (p = 0.0313) but no effect in the high contrast fixation condition (p = 1) or the low contrast fixation condition (p = 0.6875).

To further demonstrate the consistency of the effect across subjects, we chose the temporal window of maximal suppression (from 30 ms before the saccade until saccade onset) and compared percentage correct in that window for the two luminance conditions. Figure 5 shows the results and compares them to the fixation condition. All subjects' performance in the 20/20 saccade condition was better than in the 60/20 saccade condition (green squares). By comparison, the performance during fixation (black diamonds) was similar in both luminance conditions.

Because performance in the fixation condition was high, one could argue that a background luminance effect might be present at fixation but could not be found because detection was at ceiling. To verify that this was not the case, we re-ran the fixation condition with gratings whose contrast was lowered so that performance was well below ceiling. The grey circles in Figure 5 compare the subjects' performance in these control fixation conditions. Just as for the high-contrast fixation conditions, the background luminance had no significant influence on the subjects' performance. We also used these new fixation conditions together with the saccade conditions to compute the same visual sensitivity index as before and confirmed that the effect of background luminance was highly significant (p = 0.007, F = 18.71). This confirms that the cross-hemifield interaction between background luminance and detection performance was specific to the eye movement condition.

Finally, we performed two technical controls to demonstrate that the hemifield interaction could not be due to the inadvertent leakage of light through the barrier. First, we repeated the experiment for one subject wearing an eye patch over the left eye. Under these conditions, the background luminance change did not affect the suppression (not shown). Second, we measured the amount of light reflected off the physical barrier that could enter the left eye; it was below the threshold of our photometer (0.01 cd/m^2^) in both background luminance conditions.

## Discussion

Our data show that the amount of saccadic suppression in one hemifield is affected by the background luminance in the other hemifield. This hemifield interaction is not found during fixation. Given that visual information from the two hemifields is first combined in the cortex, we conclude that the mechanisms underlying the reduction of visual sensitivity around eye movements must include cortical components.

Our data do not address the question why or how contralateral changes in luminance affect saccadic suppression. These are certainly interesting questions, but they are outside the scope of our behavioral study. Two points, however, are worth making. First, the finding that background luminance affects suppression is not new. Burr et al. [5] reported this effect and related it to the sensitivities of movement systems at low luminance. We merely used this known phenomenon in a split-hemifield task to test a specific hypothesis about the involvement of the thalamus in saccadic suppression. Second, text book knowledge has it that luminance information is discarded at the retina; in this context our findings may appear mysterious. If the behavioral data of Burr et al are not enough to dispel the notion that luminance is lost at the retina, there is also direct physiological evidence. Rossi et al [8], [9] showed that the response of orientation tuned neurons in primary visual cortex are modulated by luminance changes more than 15 dva away from the classical receptive field. These extra-classical luminance modulations were not investigated in the context of eye movements, nor did these authors specifically investigate whether such modulations cross hemifields, but their data show that primary visual cortex already contains machinery by which background luminance can affect the responses to a remote visual stimulus. It does not seem a stretch to suppose that this machinery could be co-opted by the mechanisms of saccadic suppression, but only a physiological experiment could provide convincing evidence of this.

Our goal in this project was to test the hypothesis that saccadic suppression could have a purely thalamic origin. We believe that our data reject this hypothesis. Note that we do not deny that the LGN may play a role in saccadic suppression. Our data merely show that the LGN alone cannot account for the reduction in visibility around eye movements. In fact, our data do not exclude the possibility that cortex regulates the amount of suppression, while the LGN performs the actual suppression. Given the wide range of effects found with single cell recordings [10]–[13], functional imaging in cortical areas [14]–[17], and even the complexity of behavioral changes around saccades [18], however, we believe that it is unlikely that all perisaccadic response changes have the same thalamic origin. Instead, we believe that current behavioral, electrophysiological, and imaging data favor the view that saccadic suppression involves the intricate interplay of many cortical and subcortical areas.

While our data show that cortical processing plays a role in saccadic suppression, they leave open the question why cortical TMS phosphenes undergo saccadic suppression while retinal phosphenes do not [2]. One possible answer is that the details of TMS stimulation are different for retinal versus cortical phosphenes. This is the case in the purely technical sense of stimulation amplitude and shape, but also in the more qualitative sense that cortical TMS will evoke a very different pattern of feedforward and feedback neural activity [19] than retinal TMS. We speculate that such a qualitatively different pattern of neural activity makes the retinal, but not the cortical TMS phosphenes susceptible to saccadic suppression.

The mechanisms underlying saccadic suppression are a subject of ongoing debate, but in general both visual masking and an extraretinal signal undoubtedly play a role [3]. In our experiments, gratings were flashed before saccade onset, and the backgrounds were uniform. The work of Diamond et al [6] suggest that under these conditions one primarily measures the influence of an extraretinal suppression mechanisms and the influence of backward masking is small. This would suggest that the cortical components whose influence we report are mainly part of an extraretinal suppression mechanism. To completely disentangle backward masking and extraretinal components, however, future experiments would need to make use of simulated saccades.

We conclude that our study presents behavioral evidence showing that the phenomenon of saccadic suppression involves processing in higher visual cortical areas, and cannot be solely explained by changes in neural activity in the LGN.

## Methods

### Ethics Statement

This study was conducted according to the principles expressed in the Declaration of Helsinki. The study was approved by the Institutional Review Board of Rutgers University and the University of Medicine and Dentistry of New Jersey (Protocol 0120050356). All subjects provided written informed consent.

### Subjects

Six subjects participated in this study. They all had normal or corrected-to-normal vision. With the exception of one author (GC), all were naïve to the purpose of the experiment.

### Experimental Setup and Paradigm

We measured the detection of a luminance-modulated grating flashed in the right visual field during fixation and just before an eye movement in two background luminance conditions. In the first condition, the luminance was the same in both visual fields (20 cd/m^2^); we refer to this as the 20/20 condition. In the second condition, the luminance in the left visual hemifield was increased to 60 cd/m^2^ while it was kept at 20 cd/m^2^ in the right hemifield; this is referred to as the 60/20 condition. We made sure that the change of luminance was confined to the left visual field by placing a black opaque barrier that prevented the leakage of light from one hemifield to the other. Figure 1 illustrates this separation.

Each block of trials consisted of 100 trials, randomized between fixation and saccade conditions. The background luminance in each visual field was held constant in each block, but was changed between blocks.

In fixation trials, the subject fixated a red dot in the right hemifield, 4.5 degrees of visual angle (dva) to the right of the vertical midline and 3.5 dva above the horizontal midline. A sinusoidal luminance-modulated horizontal grating then appeared for 8 ms, 4 dva to the right of the fixation dot, and either 3.5 dva above or below it. The subject reported the position of the grating in each trial by pressing the appropriate keyboard button. The grating had a rectangular shape with a size of 2×5 dva, a spatial frequency of 0.15 cycles per degree. Its mean luminance was 20 cd/m^2^; equal to the background luminance.

In saccade trials, all parameters were the same except that 1500 ms after the start of the trial, the first fixation dot disappeared and a second fixation dot appeared 9 dva to the right of the first one. The subject had to make a saccade to the second fixation dot within 300 ms. The time at which the grating was flashed was adjusted per subject to ensure that most grating stimuli appeared just before saccade onset, when saccadic suppression is known to be maximal [6]. We tracked the positions of the left and right eyes using infrared cameras linked to an eye tracking system (Eyelink 2.0, SR Research, Toronto, Canada).

In a first experiment, we timed the presentation of the grating such that it arrived in a time window starting 75 ms before saccade onset, and ending with saccade onset. Restricting ourselves to this single time window left room to vary the contrast of the grating (0.5, 5, 15, 50, 90% Michelson contrast) and thereby estimate a change in perceptual threshold.

In the main experiment we wished to measure the pre-saccadic time course of detection and thus presented the grating at various times before the onset of the saccade. To allow the collection of sufficient repetitions per time window, the grating contrast was held constant at 50%. In this experiment we analyzed the percentage correct detection.

### Data Analysis

To quantify visibility of the flashed gratings, we calculated for each subject the percentage of correctly reported grating positions separately for each contrast, time window, background luminance, and saccade or fixation condition. For the analysis in Figure 2, a Weibull function was fit to the percentage correct detection as a function of contrast [7].

To quantify the specific effect of saccades and to account for the possible influence of background luminance on performance during fixation, we defined a visual sensitivity index. This index is the ratio of detection in the saccade condition and detection in the fixation condition. We calculated visual sensitivity indices from 60 ms prior to the saccade until saccade onset in non-overlapping 20 ms intervals. This calculation was done separately for each subject and for each luminance condition (Figure 3), and then averaged across subjects (Figure 4). We analyzed the population visual sensitivity indices with a two way RM ANOVA with factors of time-to-saccade and background luminance.
